# Supplementary material for: Identification of Genes Associated with Gut Microbiota Metabolites in Type 2 Diabetes Mellitus Complicated by Acute Cerebral Infarction Based on Bioinformatics and Mendelian Randomization
Source: Biomedicines. 2026 Oct 17;14(10):2371. doi: 10.3390/biomedicines14102371 (PMC13702449; doi:10.3390/biomedicines14102371)
Supplement: Supplementary file 1 [file biomedicines-14-02371-s001.zip › Figure S1/genus.Sellimonas.id.14369 T2DM.pdf]

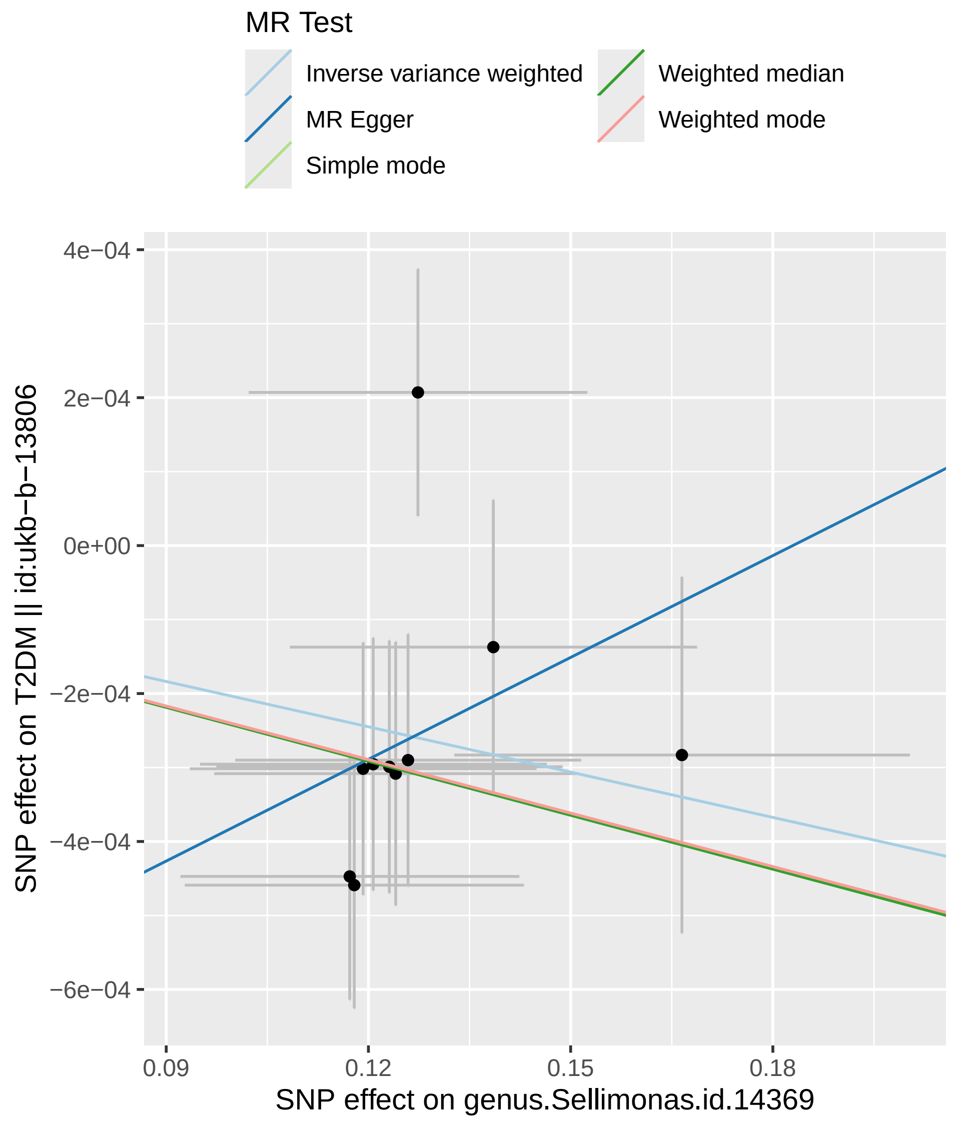

MR Test
Inverse variance weighted
MR Egger
Simple mode
Weighted median
Weighted mode
4e−04
2e−04
0e+00
−2e−04
−4e−04
−6e−04
0.09
0.12
0.15
0.18
SNP effect on T2DM || id:ukb−b−13806
SNP effect on genus.Sellimonas.id.14369
